# Phosphorus and Nitrogen Drive the Seasonal Dynamics of Bacterial Communities in *Pinus* Forest Rhizospheric Soil of the Qinling Mountains

**DOI:** 10.3389/fmicb.2018.01930

**Published:** 2018-08-27

**Authors:** Hai H. Wang, Hong L. Chu, Qing Dou, Qing Z. Xie, Ming Tang, Chang K. Sung, Chun Y. Wang

**Affiliations:** ^1^College of Forestry, Northwest A&F University, Yangling, China; ^2^Department of Food Science and Technology, College of Agriculture and Biotechnology, Chungnam National University, Daejeon, South Korea

**Keywords:** season dynamics, *Pinus*, rhizosphere soil, bacterial community, biodiversity

## Abstract

The temporal distribution patterns of bacterial communities, as an important group in mountain soil, are affected by various environmental factors. To improve knowledge regarding the successional seasonal dynamics of the mountain soil bacterial communities, the rhizospheric soil of a 30-year-old natural secondary *Pinus tabulaeformis* forest, located in the high-altitude (1900 m a.s.l.) of the temperate Qinling Mountains, was sampled and studied during four different seasons. The bacterial community composition and structure in the rhizospheric soil were studied using an Illumina MiSeq Sequencing platform. Furthermore, the edaphic properties and soil enzymatic activities (urease, phosphatase, and catalase) were measured in order to identify the main impact factors on the soil bacterial community. According to the results, all of the edaphic properties and soil enzymatic activities were significantly affected by the seasonal changes, except for the C/N ratio. Although the biomasses of soil bacterial communities increased during the summer and autumn (warm seasons), their Shannon diversity and Pielou’s evenness were decreased. Proteobacteria, Acidobacteria, Actinobacteria, Planctomycetes, and Bacteroidetes were the predominant bacterial groups in all of the soil samples, and the genera of *Ktedonobacter*, *Sphingobium* as well as an unclassified member of the Ktedonobacteria were the keystone taxa. The composition and structure of soil bacterial communities were strongly impacted by the edaphic properties, especially the temperature, moisture, ammoniacal nitrogen, available phosphorus and total phosphorus which were the crucial factors to drive the temporal distribution of the soil bacterial community and diversity. In conclusion, the soil temperature, moisture and the nutrients N and P were the crucial edaphic factors for shaping the rhizospheric soil bacterial communities as season and climate change in a *P*. *tabulaeformis* forest of Qinling Mountains.

## Introduction

Soil microorganisms perform important ecosystem services, such as improving soil structure and soil aggregation, recycling soil nutrients and water (Shen et al., 2013). Bacteria is one of the most important groups in the soil with a population ranging from 100 million to 3 billion per gram soil, comprising both the largest number and biomass of any soil microorganism (Ingham et al., 2000). Soil bacteria have been described as a bag of enzymes and fertilizer because they can reproduce quickly when optimal water, food and environmental conditions occur, even under starvation conditions or soil water stress (Dick et al., 1996). In terrestrial systems, soil bacteria play an important role as a driving force in the soil process and are very sensitive to ongoing climate changes (Schindlbacher et al., 2011). Most of soil enzymes are the microbial extracellular production. Taking soil urease and phosphatase for example, they are derived from soil microorganisms and involved in the transformation of soil N and P (Puissant et al., 2015). Therefore, soil enzymatic activities are related to the soil microbial biomass and abundance, as well as the soil community composition (Claassens et al., 2008; An et al., 2009; Siles et al., 2016). Moreover, variation in soil enzymatic activities could indicate changes in the soil metabolic capacity and nutrient cycling (Saha et al., 2008).

Baas-Becking (1934) stated a viewpoint of microbial biogeography that “everything is everywhere, the environment selects.” Therefore, soil microclimate and environmental factors, such as soil temperature, moisture, pH, nutrients, soil texture, and biotic factors, have been demonstrated to be the dominant factors and can significantly affect the distribution of microbial community (Regan et al., 2014; Shi et al., 2014). Generally, the primary direct effects of climate change on the soil microbial community activity and reproduction were supposed to be caused by changes in soil moisture, humidity, and temperature (Wang et al., 2014). Temperature affects soil microbial activity directly (Whitaker et al., 2014), and variation in soil enzymatic activities has been reported along the climatic gradients. However, there are different voices regarding to the influence of temperature on the soil microbial community. Zogg et al. (1997) suggested the soil microbial biomass increases with the global warming, but some other researchers considered it decreased (Allison and Treseder, 2008) or was not influenced (Zhang et al., 2005) when temperature increased. The changes in temperature and moisture conditions have been linked to changes in the soil microbial community composition (Carletti et al., 2009), while Corneo et al. (2014) did not find any effect of warming (about 2°C difference) on the soil bacterial community under controlled conditions. Therefore, impact of temperature on the soil microbial community is still not very clear, and more researches are necessary and required in order to understand the influence of climate change on the soil microbial community.

Microbial communities react to environmental fluctuations by adjusting their functional and species composition according to the climatic and nutritional conditions (Wallenstein and Hall, 2012). Plant species and diversity, driven by temperature and moisture content, also strongly influence the soil microbial community composition (Shi et al., 2014). Soil pH, closely correlated with the temperature and vegetation changes (Xu et al., 2014), is the most important influencing factor for driving the composition and alpha-diversity patterns (Wang et al., 2015) of soil microbial community. Seasonal change in the specific soil microclimate, specific plant activity and secretion of root exudates, and in the quality of soil microbial substrate will result in the corresponding changes in the soil enzymatic activities, the soil microbial biomass and community structures (Tan et al., 2014). For example, seasonal change directly alters the edaphic factors, such as temperature, humidity, vegetation, nutrient concentrations, and carbon (C) input, which strongly affect the soil microbial communities (Regan et al., 2014). Nemergut et al. (2010) reported the different C input could directly alter other soil nutrients (e.g., the C/N ratio) as well as the soil microbial community structure (Fierer et al., 2009).

With the global warming and the deteriorating of the climate, as well as the development of molecular tools over recent years, increasing attention has been concentrated on the study of microorganism distribution patterns across the space and time (Luo et al., 2011). High-altitude mountain soil has become an interest research area because climate change is occurring more intensely in cold regions, such as higher latitudes and altitudes (Parry et al., 2007). Study on the seasonal dynamics of soil microbial communities can improve our understanding about the influence of climate change on the soil microbial communities to some extent. Therefore, the seasonal dynamics of rhizospheric soil bacterial communities were studied recently by choosing the Qinling Mountains as the research site. The Qinling Mountains are the most important natural climatic boundary between the subtropical and warm temperate zones of China, and support an astonishingly high biodiversity. They are considered as a plant “kingdom.” The Huoditang forest region lies on the south-facing slope of the Qinling Mountains, with an annual mean temperature of 8–10°C and a frost-free period of 170 days. The annual precipitation is 900–1200 mm, and the mean evaporation is 800–950 mm, such that a high humidity of 77% occurs in the surface soil. Rainfall occurs largely from July to September, snowfall from the beginning of October through the winter and thawing at the beginning of May. Mountain brown earth is the dominant soil in this region and is classified as Eutric Regosols in the FAO (Food and Agriculture Organization of the United Nations) system, with a depth ranging from 30 to 50 cm. A vast area of natural *Pinus tabulaeformis* secondary forests is distributed from 1300 to 2300 m above sea level (a.s.l) after selective logging during the 1960s and 1970s. All of these features suggest that the Huoditang forest region in Qinling Mountains is an ideal site to study the substitution of soil microbial communities driven by seasonal change. However, there is no any report about the seasonal dynamics of soil microbial communities so far.

In this study, the rhizospheric soil samples were collected from the *P. tabulaeformis* forests located in the high-altitude of Huoditang forest region over the four seasons. Subsequently, the soil bacterial communities were analyzed by Illumina MiSeq sequencing based on the 16S rRNA gene. Moreover, the edaphic properties, such as the soil microclimate, physicochemical properties and enzymatic activities, were also measured and their correlations with the soil bacterial communities were analyzed. The objectives of this study were listed as follows: (i) to characterize the edaphic properties at the four different seasons; (ii) to make clear the seasonal dynamics of soil bacterial communities, including their activities, structures, compositions and alpha-diversities; (iii) to reveal the correlations between the seasonal dynamics of soil bacterial communities and the edaphic properties; and (iv) to make clear the influence of season and climate changes on the soil bacterial communities and find the key shifting driven factors. We assumed the season changes over the year will be significantly affect the rhizospheric soil bacterial community, and this influence is performed via changing the soil microclimate factors and physicochemical properties. In addition, certain nutrients may be instead of the soil organic matter to become the limiting factors for the seasonal dynamics of soil bacterial communities, since there is large quantity of plant humus on the temperate forest floor.

## Materials and Methods

### Sample Collection and Preparation

Three natural secondary *P*. *tabulaeformis* forests, located at the Huoditang forest region of Qinling Mountains (approximately 1900 m a.s.l., latitude 33°18′–33°28′N, and longitude 108°21′–108°39′) in Ningshan county, Ankang city, Shaanxi Province, were selected for this study. The rhizospheric soil samples were collected during summer (August 16, 2014), autumn (November 3, 2014), winter (January 20, 2015), and spring (May 8, 2015), respectively. To investigate the soil bacterial shift dynamics with seasons, *P*. *tabulaeformis* individuals with similar characteristics were selected to avoid differences caused by other factors. Five trees per study forest were selected and they were separated from each other at least 10 m apart. After removing the forest floor, three 0–30 cm soil cores with a diameter of 4 cm were taken from each tree by using a soil corer to collect the rhizospheric soil (soil adjacent to the whole root system) samples. All of the sampling sites were 1.5 m away from the base of the tree and approximately equidistant from each other. Three rhizospheric soil samples from the same tree were pooled together and separately placed into the polythene seal bags, and then were placed into an ice box and transported to the laboratory within 2 days. In total, 60 soil samples were collected over the four seasons. Root and plant material in soil samples were discarded before post-processing. After homogenizing and sieving through a 1 mm mesh, each soil sample was separated into two parts: one was stored in a freezer at -80°C for the nucleic acid analysis, and another was air dried at room temperature for the analysis of soil physical-chemical parameters.

### Edaphic Properties Analysis

Soil moisture content (SMC) was calculated as (soil fresh weight – dry weight)/fresh weight × 100%, in which soil dry weight was determined by drying soil samples at 105°C to a constant weight (Guan et al., 1986). Soil temperatures (STs) were detected during the sampling process by using an angle stem earth thermometer at depths of 5, 10, 15, 20, and 25 cm to calculate the mean value. Three points were measured at each sampling forest. Soil pH was measured in a 1:5 (w:v) deionized aqueous solution using a pH meter, which was regularly calibrated during the test process (PHS-3C; Shanghai Precision & Scientific Instrument, Shanghai, China).

The soil physical-chemical parameters were measured by using the air-dried soil samples, and each treatment was conducted in quadruplicate. Soil total nitrogen (TN), total phosphate (TP), and total potassium (TK) were measured by inductively coupled plasma atomic emission spectrometry (ICP-AES) after being digested by HNO_3_ + HClO_4_. Ammonium nitrogen (NH_4_^+^) and nitrate nitrogen (NO_3_^-^) were extracted by KCl and measured by SEAL Auto Analyzer 3 (AA3, Germany), Available K (AK) was extracted with NH_4_OAc and then measured by ICP-AES; and available P (AP) was extracted by NH_4_HCO_3_ + DTPA (diethylenetriamine-pentaacetic acid) and measured by ICP-AES. Soil organic matter was measured using the potassium dichromate volumetric method (Tan, 2005). Soil catalase activity was measured using the KMnO4 titrimetric method; phosphatase activities were determined by p-Nitrophenyl Phosphate Disodium method (pNPP); urease activity was determined using the colorimetric determination of ammonium (Guan et al., 1986).

### Soil Microbial DNA Extraction, PCR Amplification and Sequencing

The microbial DNA was extracted from 0.5 g of the rhizospheric soil samples using the E.Z.N.A.^®^ Soil DNA Kit (Omega Bio-tek, United States) following the manufacturer’s instructions. After the template DNA being quantified, PCR reactions were performed targeting the V4–V5 regions of the bacterial 16S rRNA gene using a BIO-RAD IQ5 thermal cycler (Bio-Rad Laboratories, Inc., CA, United States) to determine the bacterial abundance. Primer pair 515F (5′-barcode-GTGCCAGCMGCCGCGG-3′) and 907R (5′-CCGTCAATTCMTTTRAGTTT-3′) (Rösch et al., 2002) was used for this amplification, in which barcode is an eight-base sequence unique to each sample. The PCR mixture was prepared in a final volume of 20 μL, containing 4 μL of 5 × FastPfu Buffer, 2 μL of 2.5 mM each of dNTP, 0.4 μL of each primer (5 μM), 0.4 μL of FastPfu Polymerase (TIANGEN Biotech Co., Ltd, Beijing, China), 10 ng of template DNA, and then ddH_2_O up to 20 μL. PCR was conducted with an initial denaturation step at 95°C for 2 min, followed by 25 cycles of denaturation at 95°C for 30 s, annealing at 55°C for 30 s, and elongation at 72°C for 30 s, and then a final elongation step at 72°C for 5 min. PCR products were checked with 2% agarose gel and purified using the AxyPrep DNA Gel Extraction Kit (Axygen Biosciences, Union City, CA, United States) according to the manufacturer’s instructions. Furthermore, five purified amplicons from the same sampling forest during each season were pooled together to provide one representative equimolar amplicon library. Therefore, twelve amplicon libraries were obtained in total. Finally, Illumina MiSeq sequencing was performed, and an Illumina MiSeq platform (Majorbio, Shanghai, China) was used in this study according to standard protocols (Caporaso et al., 2012).

### Sequence Data Processing

Before the following analysis, primers were removed from the obtained sequences. In addition, the sequence with a length <200 bp, the read average Phred score <25 or with any mismatches in primer or tag sequences were removed. Furthermore, reads with low-quality regions were also discarded. Reads and quality scores were filtered using QIIME v. 1.6.0 (Caporaso et al., 2010). To maintain a sequencing depth consistent across all samples, random subsampling of 17,220 reads was conducted from per sample before downstream analyses. Denoised sequences were clustered into operational taxonomic units (OTUs) after removing single reads using UPARSE (version 7.1^1^) with a 97% similarity cut-off (Edgar et al., 2011). In each OTU, the most abundant sequence was selected as the representative sequence. Taxonomic classification of representative sequences from an individual OTU was performed by blasting against the Silva (Release119^2^) SSU rRNA database with a confidence threshold of 70% (Quast et al., 2012) using an RDP Bayesian classifier (v.2.2^3^) on the QIIME platform.

### Statistical Analysis

Alpha diversity and beta diversity based on Bray-Curtis distance were calculated using 17,220 reads per sample (minimum number of sequences required to normalize the differences in sequencing depth) by Vegan packages in R. Principal component analysis (PCA) was performed using soil physiochemical properties as variables, and alpha diversity indexes (or genus relative abundance) were fitted as factors onto the ordination. The genus presence/absence data for soil bacteria was visualized using non-metric multidimensional scaling (NMDS) to elucidate the dissimilarities in bacteria community composition across the sample sites along the chronosequence (Oksanen et al., 2015). PCA and NMDS were performed in an R environment using a Vegan package.

The intergroup differences in genus were obtained in R using a Kruskal-Wallis rank sum test, and the genus with *P* < 0.05 were selected for post-analysis. To examine co-occurrence networks in bacterial communities, network analysis was conducted based on the edaphic properties and selected genera by using Spearman’s correlation analysis in SPSS (version 20.0; SPSS, Chicago, IL, United States). A Spearman’s correlation between two genera was considered statistically robust when the Spearman’s correlation coefficient (ρ) was >0.6 and the *P*-value was <0.01 (Barberán et al., 2012). The networks were then visualized in CYTOSCAPE version 3.2.0 (Shannon et al., 2003). A total of 100 nodes (88 biotic variables and 12 edaphic factors) and 844 edges were generated in the co-occurrence network. To assess the non-random pattern in the resultant network, 10,000 Erdös-Rényi random networks were generated in order to compare with the topology of the real network, with each edge having the same probability of being assigned to any node. Network topology parameters were calculated using the *Network Analyzer* tool. To describe the topology of the resulting networks, a set of measures (number of nodes and edges, average path length, network diameter, average degree, graph density, clustering coefficient, and modularity) was calculated. Keystone species were defined as the genera with the highest betweenness centrality value, which indicate the relevance of a node as capable of binding together communicating nodes (González et al., 2010; Vick-Majors et al., 2014). Modular structure and groups of highly interconnected nodes were analyzed using the MCODE application with standard parameters (degree cut-off of 2, node score cut-off of 0.2, K-core of 2 and maximum depth from seed of 100) (Bader and Hogue, 2002).

A structural equation model (SEM) was performed to detect the causal relationships among the edaphic properties, bacterial community composition, alpha-diversities and enzymatic activities by using AMOS 17.0 (Amos Development Corporation, Meadville, PA, United States). Mantel tests were conducted with the “mantel” function in the Ecodist package (Goslee and Urban, 2007) to examine the interrelationships among the edaphic variables, rhizospheric soil bacterial characteristics (community composition and alpha-diversities) and soil enzymatic activities (urease, phosphatase, and catalase). The *r* values derived from the mantel tests were considered as input data to create the SEM. Maximum-likelihood estimation was applied to compare the SEM model with the observation. Values of *χ*^2^ tests, goodness-of-fit index (GIF), Akaike information criteria (AIC) and root mean square error of approximation (RMSEA) were used to determine the model adequacy. Adequate model fit was indicated by a non-significant *χ*^2^ test (*P* ≥ 0.05), high GFI (>0.90), low AIC and low RMSEA (<0.05). All statistics analyses were performed in an R environment unless other indication.

## Results

### Characteristics of Rhizospheric Soil Samples

All of the measured soil physico-chemical parameters showed high significant variability among the different seasons (*P* < 0.05), except for the C/N ratio (*P* ≥ 0.5) (**Table 1**). SMC was the lowest in winter (13.4%), increased from spring to autumn, and reached the highest in autumn (*P* < 0.001). ST changed along with the seasons and was directly influenced by the air temperature (*P* < 0.001), but all of them stayed above the freezing point even in winter. All of the soil samples were acidic with the pH values ranged from 4.88 to 6.08 (*P* < 0.001). The highest pH value was showed in spring and the lowest one in summer and winter. The contents of TK and NO_3_^-^ reached the highest value (*P* < 0.001) in spring and decreased continuously until winter. As to the contents of TN (*P* < 0.05), AK (*P* < 0.001), and SOM (*P* < 0.05), they exhibited the highest value in the autumn and winter, followed by spring, and the lowest in summer. The content of TP reached the highest value (0.579 g/kg) in spring, followed by winter, and decreased to the lowest in the warm seasons (summer and autumn). However, the concentration of AP (*P* < 0.001) and NH_4_^+^ (*P* < 0.05) has the lowest values in spring, then increased continuously and touched the highest value in autumn.

**Table 1:** Environment factors of sampling sites.

Environmental factor	Season				*F*	*P*
	Spring	Summer	Autumn	Winter		
SMC (%)	0.161 ± 0.002b	0.187 ± 0.008b	0.283 ± 0.002a	0.134 ± 0.002c	89.024	^∗∗∗^
ST (°C)	4.72 ± 0.03c	16.72 ± 0.01a	8.65 ± 0.03b	1.23 ± 0.03d	23991	^∗∗∗^
pH	6.10 ± 0.09a	4.96 ± 0.02c	5.2 ± 0.04b	4.88 ± 0.07c	34.47	^∗∗∗^
TK (g/kg)	4.525 ± 0.003a	3.437 ± 0.20b	2.738 ± 0.072c	2.642 ± 0.072c	42.45	^∗∗∗^
TN (g/kg)	2.457 ± 0.015b	2.19 ± 0.17b	2.933 ± 0.001ab	3.342 ± 0.127a	4.18	^∗^
TP (g/kg)	0.579 ± 0.003a	0.22 ± 0.007c	0.2 ± 0.006c	0.351 ± 0.005b	25.47	^∗∗∗^
AP (mg/kg)	5.785 ± 0.169c	10.358 ± 1.03b	14.32 ± 0.665a	11.381 ± 0.562b	14.975	^∗∗∗^
AK (mg/kg)	89.382 ± 4.387b	50.446 ± 1.912c	116.728 ± 0.657a	117.55 ± 0.609a	11.39	^∗∗∗^
NO_3_^-^(mg/kg)	9.81 ± 0.885a	3.357 ± 0.182b	3.405 ± 0.088b	1.791 ± 0.077c	48.95	^∗∗∗^
NH_4_^+^ (mg/kg)	17.086 ± 0.3b	21.839 ± 1.805ab	27.569 ± 1.133a	19.866 ± 0.077b	3.25	^∗^
SOM (g/kg)	42.214 ± 1.133ab	40.13 ± 5.47b	49.526 ± 0.338ab	56.868 ± 4.367a	2.903	^∗^
C/N	10.176 ± 0.295ab	10.334 ± 0.592a	9.875 ± 0.058ab	8.586 ± 0.308b	2.357	NS


The data represent the mean ± SD. Different small letters indicate significant difference among different seasons on that parameter. Means are compared using one-way ANOVA at *P* < 0.05 level. ^∗^Significant at *P* < 0.05; ^∗∗^ significant at *P* < 0.01; ^∗∗∗^Significant at *P* < 0.001; NS, no significant difference.

**FIGURE 1:**
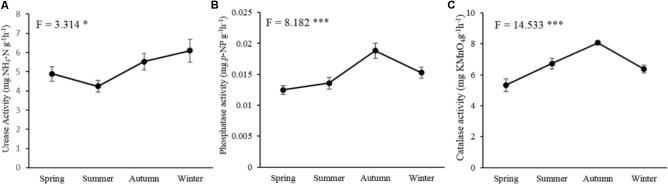
Seasonal changes of the soil microbial enzymatic activities in *P*. *tabulaeformis* forest located on Qinling Mountains. **(A)** urease activity; **(B)** phosphatase activity; and **(C)** catalase activity. Error bar denotes standard error. ^∗^*P* < 0.05, ^∗∗^*P* < 0.01, ^∗∗∗^*P* < 0.001.

The tested three soil enzymatic activities, including urease (*P* < 0.05), phosphatase (*P* < 0.001), and catalase (*P* < 0.001), were significantly affected by seasonal changes (**Figure 1**). The activity of urease showed the highest value in winter and decreased in spring and summer (**Figure 1A**). Phosphatase (**Figure 1B**) and catalase activities (**Figure 1C**) increased in the warm seasons with the highest value in autumn, but decreased in the cold seasons.

### Distribution of Taxa

After being normalized and optimized, 17,220 quality sequences were obtained from each rhizospheric soil sample and used for the subsequent analysis, so that 206,640 sequences were produced in total (*n* = 12). At the 97% similarity threshold, 904 OTUs were obtained and identified as 277 genera, 23 bacterial phyla. Among these phyla, Nitrospirae, Chloroflexi, Gemmatimonadetes, Verrucomicrobia, Firmicutes, Elusimicrobia, Cyanobacteria, Chlorobi, and Armatimonadetes were detected at a lower relative abundance, while Proteobacteria, Acidobacteria, Actinobacteria, Planctomycetes, and Bacteroidetes were predominant (relative abundance >5%) and accounted for 88.2% of the total sequences. The phylum Proteobacteria, with a relative abundance of 41.94%, was represented by the following classes: Alphaproteobacteria (24.71%), Betaproteobacteria (8.51%), Gammaproteobacteria (5.14%), and Deltaproteobacteria (3.04%). In addition, the phylum Acidobacteria (29.14%) was represented by the class of Acidobacteria (27.78%).

**FIGURE 2:**
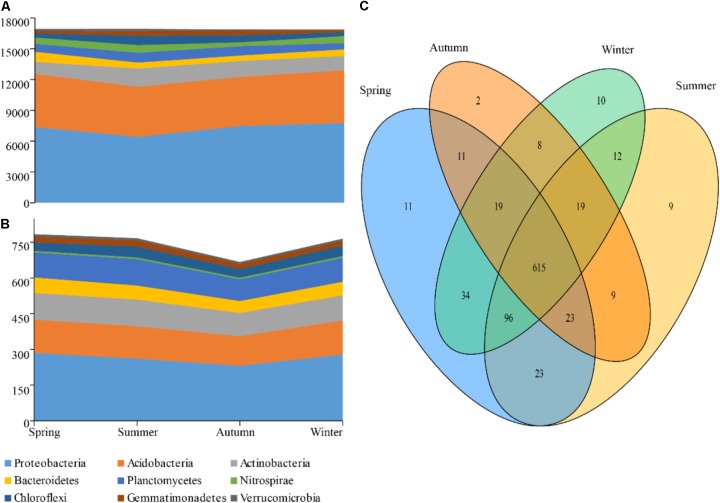
Taxa dynamics and Venn diagram of soil bacterial communities. The reads number **(A)** and OTUs **(B)** that are present and shared in at least 0.5% of the population at indicated seasons are aggregated and colored by phylum on a stream-graph; **(C)** Venn digram showing the different OTU numbers between each two seasons.

The reads numbers and OTUs numbers of phyla were compared season by season (**Figure 2**). According to the results, no marked difference was observed in the reads number of Acidobacteria throughout the seasons. In summer, however, the reads numbers of Proteobacteria and Bacteroidetes had a pronounced constriction, while an expansion was showed in those of Actinobacteria and Planctomycetes (**Figures 2A,B**). Moreover, a total of 615 OTUs were detected as the common species of four different seasons, while 55 OTUs as the endemic species were found in the cold seasons and 20 OTUs in the warm seasons (**Figure 2C**). The OTUs numbers of phyla showed an obvious decrease in autumn with only two special OTUs.

**FIGURE 3:**
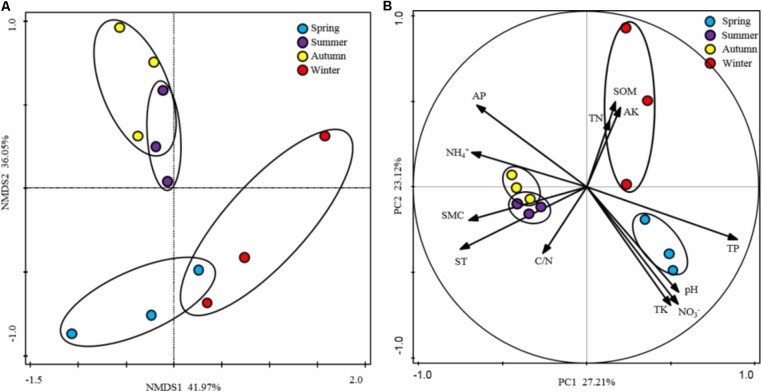
Ordination analysis of the variation in rhizospheric soil bacterial communities. **(A)** Non-metric multidimensional scaling (NMDS) plots of the variation in the rhizospheric soil bacterial communities of *P*. *tabulaeformis* forest based on the profiles of presence/absence of genus; **(B)** Principal component analysis (PCA) of the rhizospheric soil bacterial communities of *P*. *tabulaeformis* forest based on the genus abundance and soil physicochemical characteristics as variables.

To show the variability in rhizospheric soil samples, PCA analysis fitted with the generic relative abundance (**Figure 3B**) and NMDS analysis fitted with the presence/absence (**Figure 3A**) of the genus in the rhizospheric soil bacterial communities were performed. According to the results, soil samples from the warm seasons were clustered together and showed strong homogeneity, however, soil samples from winter and spring intersected with each other. These results indicated that the composition and structure of soil bacterial community were strongly affected by the seasonal changes. Furthermore, the edaphic properties were fitted as variables in the PCA analysis to explore their influence on the soil bacterial community composition. Differences between warm seasons and cold seasons were shown in the first axis, explaining 27.21% of the total variation. Moreover, the edaphic properties of AP, NH_4_^+^, ST, SMC, and TP as the main driving factors strongly impacted the bacterial community compositions. In addition, component two explained 23.12% of the total variation, in which separations between winter and spring were shown, driven by pH, NO_3_^-^, SOM, AK, and TK.

All of these results indicated that the edaphic properties of AP, NH_4_^+^, and TP and the microclimatic factors of ST and SMC played crucial roles in shaping the rhizospheric soil bacterial communities along with the seasonal changes.

### Rhizospheric Soil Bacterial Diversity

**FIGURE 4:**
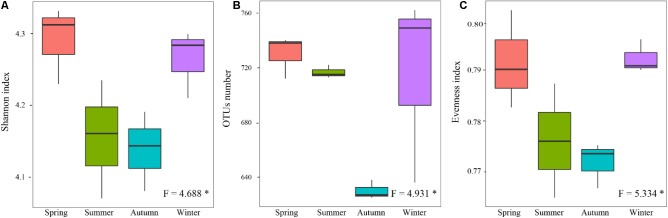
Seasonal changes in alpha-diversity of the rhizospheric soil bacterial community in *P*. *tabulaeformis* forest located on Qinling Mountains. **(A)** Shannon; **(B)** OTU richness; and **(C)** Evenness. The horizontal bars within boxes represent the median. The tops and bottoms of boxes represent 75th and 25th quartiles, respectively. The upper and lower whiskers extend 1.5× the interquartile range from the upper edge and lower edge of the box, respectively. ^∗^*P* < 0.05, ^∗∗^*P* < 0.01, and ^∗∗∗^*P* < 0.001.

Alpha-diversity indexes of the rhizospheric soil bacterial communitie, including Shannon (**Figure 4A**), OTUs richness (richness) (**Figure 4B**), and Pielou’s evenness (evenness) (**Figure 4C**), showed significant differences among four different seasons (*P* < 0.05). They showed lower values in the warm seasons compared to the cold seasons, and the lowest values presented in autumn. It seemed that their seasonal variations were following a U-shaped trend with started from the warm seasons.

**FIGURE 5:**
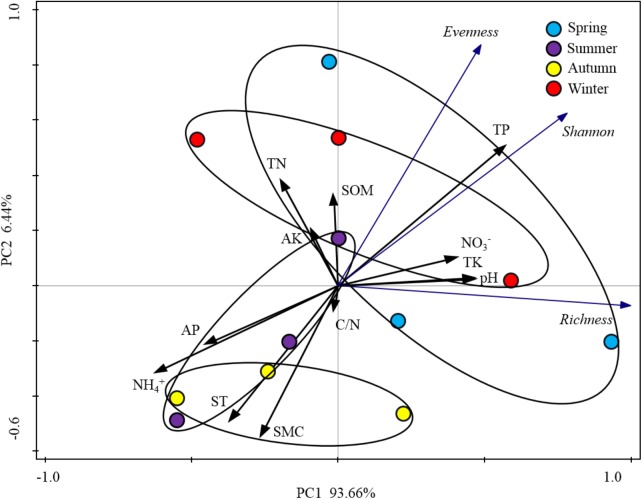
Principal component analysis (PCA) based on the soil physicochemical characteristics of *P*. *tabulaeformis* forest as variables. Alpha-diversity indices were fitted as factors with significance <0.05 onto the ordination.

When the alpha-diversities of the rhizospheric soil bacterial community were fitted as factors onto the ordination of PCA analysis, the first axis represented 93.66% of the total variability and the last 6.44% was for the second axis (**Figure 5**). Based on the edaphic factors, PCA analysis showed the rhizospheric soil samples were clustered together depending on the warm seasons and cold seasons. These results indicated that the alpha-diversity indexes of rhizospheric soil bacterial community were significantly responsive to the seasonal changes. Among the edaphic properties, SMC, AP, ST, and NH_4_^+^ were negatively correlated with the alpha-diversities, but pH, NO_3_^-^, TP, and TK showed a positive correlation. The significant correlations between the edaphic properties and alpha-diversity indexes of rhizospheric soil bacterial community were confirmed by their linear regression relationships and Spearman’s correlation test (**Supplementary Table S4**). From the Spearman’s correlation test, it could be seen the edaphic properties of TP, NH_4_^+^, ST, and SMC significantly affect Shannon, richness and evenness of the rhizospheric soil bacterial community (*P*-value was < 0.05) (**Supplementary Figure S1**).

### Networks and Connectedness

**FIGURE 6:**
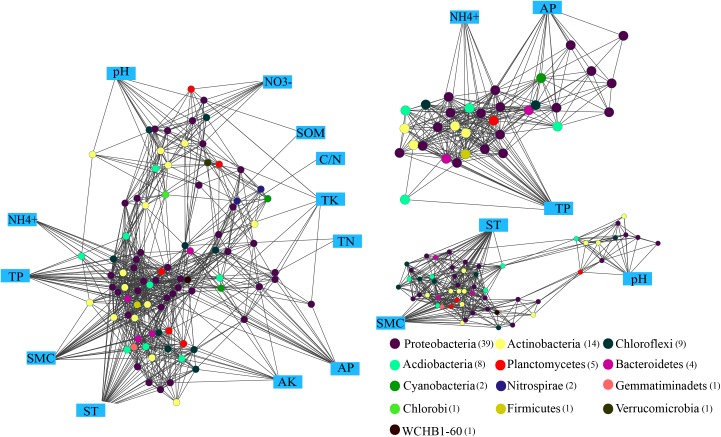
Network analysis showing the connectedness between the bacterial communities and edaphic factors in *P*. *tabulaeformis* forest located on Qinling Mountains. A connection stands for a strong (Spearman’s ρ > 0.6) and significant (*P* < 0.01) linear relationship. Variables in blue boxes represent various edaphic properties. The maximum connectedness, exhibited on the associations between the edaphic properties (AP, TP, and NH_4_^+^) and soil microclimate (ST and SMC) as well as soil pH, are shown separately.

The Kruskal-Wallis rank sum test revealed a total of 88 genara were significantly different among four seasons (*P* < 0.05) (**Supplementary Figure S2**), and 832 edges were obtained from Spearman’s correlation analysis (Spearman’s correlation coefficient (ρ) was >0.6 and the *P*-value was <0.01). The networks (**Figure 6**) were conducted with an average number of neighbors of 16.88, a characteristic path length of 2.291, a network diameter of four edges, an average clustering coefficient of 0.562 and a network density of 0.171 (**Table 2**). To describe the complex pattern of the inter-relationship among nodes and compare the real network with an identically sized Erdös-Rényi random network, significant topological properties of networks were calculated. The real-world network’s structural properties were greater than the Erdös-Rényi random network. In other words, the real network was more significantly clustered than the random network, and highly connected genera composited the microbial network and formed a “small world” topology.

**Table 2:** Topological properties of co-occurring networks.

	Clustering coefficient (CC)	Average path length (APL)	Network diameter (ND)	Graph density (GD)
Whole network	0.562	2.291	4	0.171
Random network	0.17 ± 0.01	1.87	3 ± 0.01	0.171


Random network is an identically sized random network generated based on Erdös-Rényi model, whose topological properties are calculated as the average value from 10,000 Erdös-Rényi random networks.

Genus of *Ktedonobacter*, *Sphingobium* and an unclassified member of the Ktedonobacteria were identified as the keystone species based on the betweenness centrality scores. Eight significant clusters with the network scores ranged from 16.762 to 3.0 were returned from MCODE analysis (**Supplementary Figure S3**). TP and NH_4_^+^ produced a rank 1 cluster with 22 nodes and 176 edges. A rank 2 cluster was produced from ST and SMC with 14 nodes and 54 edges, while a rank 3 cluster was consisted of only bacterial interactions. Among the edaphic properties, ST, SMC, and TP were associated with the maximum number of nodes, and followed by NH_4_^+^ and AP. Moreover, AP, TP, and NH_4_^+^ were mostly associated with nodes belonged to Proteobacteria and Actinobacteria, suggesting these three edaphic properties significantly influence the abundances of relative genera and the structures of the rhizospheric soil bacteria community.

### Relationships Between Bacterial Community Characteristics and Edaphic Properties

**FIGURE 7:**
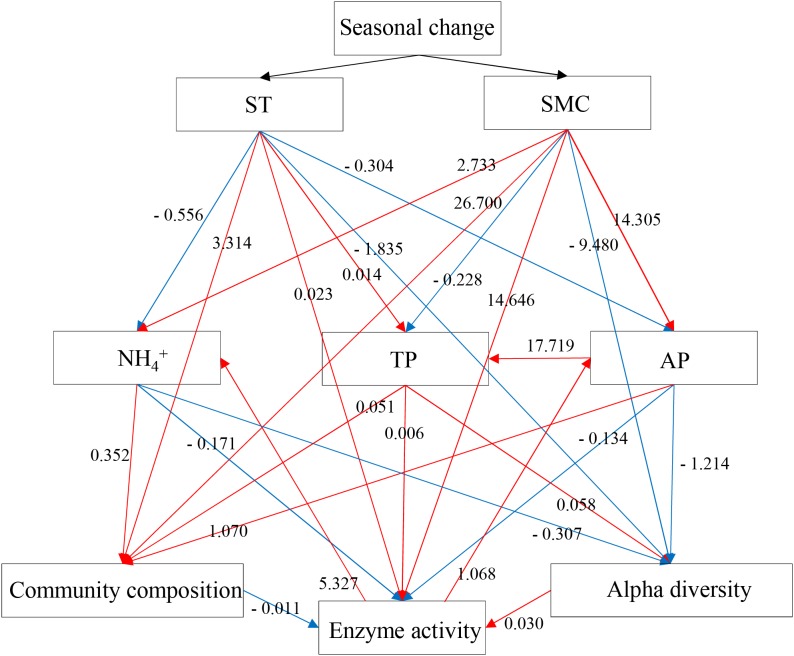
Structural equation model (SEM) showing the causal relationships among the soil bacterial community composition, alpha-diversities, enzymatic activities and edaphic properties (ST, SMC, TP, AP, and NH_4_^+^) in *P*. *tabulaeformis* forest based on the results of Mantel statistics. The final model fits the data well: maximum likelihood, *χ*^2^ = 0.1, df = 2, *P* = 0.948, goodness-of-fit index = 0.998, Akaike information criteria = 68.106 and root mean square error of approximation = 0. Red lines mean the positive standardized regression weights and blue lines indicate the negative standard regression weights.

To validate the relationships between the rhizospheric soil bacterial communities and edaphic properties, a mantel test and SEMs were performed to show the causal relationships between the variables. Mantel tests showed the soil bacterial community composition was significantly related to ST, AP, TP, and pH (**Supplementary Table S2**), while the soil enzymatic activities were significantly related to AP, TP and the soil bacterial community composition. Subsequently, the final SEM model with a Mantel *r* value as input adequately fitted the data describing the interaction pathways among the soil bacterial community composition, alpha-diversity, soil enzymatic activities and edaphic properties (ST, SMC, TP, AP, and NH_4_^+^), with values of *χ*^2^ = 0.106, df = 2, *P* = 0.948, GFI = 0.998, AIC = 68.106, and RMSEA = 0 (**Figure 7**). SEM analysis showed that the concentrations of NH_4_^+^, TP, and AP in soil samples were affected by the seasonal changes of SMC and ST, and further was to impact the soil bacterial community composition, alpha-diversity and soil enzymatic activity. Soil enzymatic activity was directly or indirectly affected by the edaphic properties and the soil bacterial community, in turn, was to impact the concentration of AP and NH_4_^+^. These results indicated that soil NH_4_^+^, AP, and TP significantly influenced the composition and alpha-diversity of the rhizospheric soil bacterial community and, in turn, soil enzymatic activity. In addition, seasonal changes in the structure and composition of soil bacterial community influenced the soil microbial extracellular enzymes, then, the process of substrate decomposition, such as soil N and P.

## Discussion

### Effects of Seasonal Change on the Edaphic Properties and Soil Enzymatic Activities

Soil temperature and moisture are directly linked to the air temperature, humidity and precipitation at different seasons (Siles et al., 2016), so that soil temperature and moisture are significant different between seasons, and have been recognized as important factors to drive the soil microbial community and soil physicochemical properties over 1 year (Zhou et al., 2015). In addition, the vegetation and alternation of vegetation period and dormant period affected also by the seasonal changes in climate, and had significant impacts on the loss and replenishment of soil nutrients (Fitzhugh et al., 2003). Kreyling et al. (2012) reported the concentration of soil organic matter changed seasonally due to litter input and the microbial degradation activity, as well as other nutrients, such as N and P cycling. In this study, the seasonal variations of climate in the *P. tabulaeformis* rhizospheric soil of the Qinling Mountains were expressed by the temporal changes in the edaphic properties, as expected. The concentrations of available nutrients, such as AP, AK, and NH_4_^+^, significantly increased in autumn, but decreased in winter (AP, NO_3_^-^, and NH_4_^+^). The accumulation of available nutrients in autumn might be related to the plant senescence and microorganism degradation (Bardgett et al., 2005; Jefferies et al., 2010), while the decrease in available nutrients in winter possibly caused by the utilization of microbial communities which stay active under the snow cover even during winter (Lipson et al., 2000).

Soil enzymatic activities provided indications of variation in metabolic capacity of the soil and in nutrient cycling (Saha et al., 2008), and were related to the soil microbial abundance and community composition (An et al., 2009). Measurement of the soil enzymatic activities in our study was expected to represent the soil process and microbial activity. As is known, the activities of soil urease and phosphatase are derived from soil microorganisms and involved in the transformation of soil N and P; whilst, soil catalase is produced as a protection against oxidative stress by the decomposition of H_2_O_2_ (Cavalcanti et al., 2004) and its activity could be used as a method to assay the microbial biomass in soil (Alef and Nannipieri, 1995). According to the previous researches, soil enzymes had higher potential activities in the warm seasons due to the increasing of microbial biomass (Jing et al., 2014). Moreover, the competition between plants and microorganisms for resource during the growing season also could lead to increased enzyme production by soil microorganism (Yao et al., 2011). Therefore, the continuous increases of phosphatase and catalase activities during warm season with a peak in autumn were possibly due to the increasing of microorganism biomass in our study. Surprisingly, our study revealed the enzyme pool size of urease increases in winter and decreased in summer. The biological activity of enzyme reduced with the declining of temperature, thus, microbes increased the enzyme production to compensate for the lower enzyme reaction kinetics at low temperature in winter (Bell et al., 2010). These explanations seemed to be confirmed in our study and helpful for understanding the different seasonal patterns of soil enzymatic activities.

### Effects of Seasonal Change on the Characteristics of Rhizospheric Soil Bacterial Community

In this study, the phyla of Proteobacteria and Acidobacteria were dominant in the rhizospheric soil bacterial communities, followed by Actinobacteria. Similar results have been reported based on the researches on other mountain forest rhizospheric soil (Rasche et al., 2011; Dai et al., 2018). Some species of Proteobacteria and Actinobacteria were reported as the copiotrophic bacteria and exhibited the opposite variation pattern, whilst, some species of Acidobacteria were tended to be more fastidious oligotrophic bacteria (Fierer et al., 2007). These could be used to explain our results that the reads of Proteobacteria decreased in summer and peaked in winter, while inverse trend was found for Actinobacteria. Moreover, no significant difference was found from the abundance of Acidobacteria over four seasons. In addition, Leff et al. (2015) suggested that N inputs inhibited the growth of oligotrophic bacteria, such as some Acidobacteria, which have the potential capability to decompose nutrient poor and recalcitrant substrates, but promoted the growth of copiotrophic bacteria (Fierer et al., 2012). Consistently, our results showed that the concentration of NH_4_^+^ was negatively correlated with the relative abundance of Acidobacteria, however positively linked with Actinobacteria. In the warm seasons, OTUs numbers of the soil bacteria were observably decreased, but the biomasses of the soil bacteria showed continuously increase. It was suggested that increase of temperature led to a remarkable increase in soil microbial biomass, but a dramatically decline in soil bacterial species richness. *Acidobacteriales_*uncultured, *Holophaga*, and *Bradyrhizobium* were the most abundant genara in the rhizospheric soil of *P*. *tabuliformis*. They have been reported to participate in the degrading of soil organic matter, methoxylated aromatic compounds and symbiotic nitrogen fixing, respectively (Banerjee et al., 2016). Because of the usefulness of betweenness centrality in disentangling the most influential taxa (González et al., 2010; Lupatini et al., 2014), network analysis in this study showed *Ktedonobacter*, *Sphingobium* and an unclassified member of the Ktedonobacteria were the most influential genera in the rhizospheric soil of *P*. *tabuliformis* and were considered as keystone taxa in the network.

As to the alpha-diversity indexes of the soil bacterial communities in this study, they were strongly affected by the seasonal changes and this affection was performed via the seasonal variation of edaphic properties. This result was consistent to the previous study at site with similar seasonal characteristics (Björk et al., 2008). Naeem (2009) showed that increase in the soil microbial diversity could lead to the raising of soil microbial biomass, since the diverse microorganism communities could use nutrient resources more efficiently through resource complementarity. However, there was a higher microbial biomass (soil catalase activity) in the rhizospheric soils of this study, but a lower diversity, richness and evenness in the warm seasons. The reductions in species richness and diversity of soil bacteria might be caused by their strong competition in nutrients. Männistö et al. (2006) suggested that soil temperature and moisture, which directly affected by the climatic changes, had a greater influence on alpha-diversity of the soil bacterial community than pH did. This study also proved this point. Previous study indicated that edaphic properties, especially TP, AP, and NH_4_^+^ content, are strongly impacted by the alpha-diversity of the soil bacterial community (Urbanová et al., 2015). Variation in the structure and composition of soil bacterial community led to changes in soil nutrient decomposition due to the different substrates of various microflora (Siles et al., 2016). Although previous studies have showed the soil microbial community diversity is significantly correlated with C or C/N ratio (Shen et al., 2013), opposite results were found in our study. This might be on account of the contents of soil litter and organic matter, which were so high that their limiting effects on the bacterial community were reduced across four seasons.

Differences in microbial community structure between sampling dates on seasonal basis in a variety of ecosystems have been found (Koranda et al., 2013). In general, microclimate changes were considered as the drive factors which affect the structure and composition of soil bacterial communities via changes in the soil chemical properties (Brockett et al., 2012). In accordance with the seasonal variation of the relative abundance of rhizospheric soil bacterial phyla, soil abiotic factors change along with the seasonal variation in this study and could impact the activity and amount of different soil bacterial populations. Changes in the soil microbial population abundance was supposed to cause a difference in preferred substrates, which significantly affect the soil nutrient content and cycling (Puissant et al., 2015). Previous studies have indicated that pH, carbon availability and C/N ratio in the soil are the most important factors for changing the soil bacterial community composition (Bartram et al., 2014; Geml et al., 2014). Moreover, variation in C source availability of root secretion was noted as one of the important factors responsible for the seasonal changes in soil microbial activity and biomass (Griffiths et al., 2003). Whereas, different result was obtained in this study that the contents of soil P and N obviously affect the soil bacterial community over four seasons. This result seemed agree with some other researches on the forest ecosystems, which showed the contents of soil available P (DeForest et al., 2012; Zhang et al., 2013) and N (Lejon et al., 2007) could affect the compositions of soil microbial communities.

In this study, the rhizospheric soil samples from the warm seasons or cold seasons were homogenous individually based on the composition of soil bacterial community. It was suggested that the soil microclimate factors (ST and SMC), determined by the seasonal variation, were seems to be the main impact factors on the composition and structure of soil bacterial community. Moreover, nutrients P and N were significantly correlated with the composition and genera abundances of soil bacterial community, indicating they were the limiting factors in shaping the rhizospheric soil bacterial community in *P*. *tabuliformis* forests of Qinling Mountains. The important roles of ST, SMC, N, and P in driving the seasonal dynamics of rhizospheric soil bacterial community and soil enzymatic activities were confirmed by SEM.

### Connectedness and Modular Structure of the Rhizospheric Soil Bacterial Communities

Networks could explore the roles of deterministic ecological processes and possible historical effects in shaping the soil bacterial communities and reveal overall co-occurrence patterns (Banerjee et al., 2016). Networks in this study demonstrated the non-random co-occurrence and connectedness of the rhizospheric soil bacterial communities across seasons. Modular structure of the rhizospheric soil bacterial communities was found in this study, showing the importance of studying on bacterial modules in the rhizospheric soil ecosystems to identify the bacterial associations across seasons and to improve our understanding on bacterial relationships with the edaphic properties. Kruskal-Wallis rank sum tests showed the most of seasonal differential genera are belonged to phyla of Proteobacteria and Actinobacteria in this study. *Ktedonobacter*, *Sphingobium* and an unclassified member of the Ktedonobacteria were identificated as the top three species based on the betweenness centrality score, suggesting they may play critical roles in maintaining the structure and function of rhizospheric soil bacterial communities. To discern the modules maintaining the connectivity of a network, betweenness centrality has highlighted its usefulness in defining the keystone species in the system (Vick-Majors et al., 2014; Banerjee et al., 2016). Previous study has indicated the node in different modules performs different functions (Newman, 2006). The network analysis supported the notion that bacterial community assembly is determined by the environmentally driven functional characteristics but not phylogeny (Burke et al., 2011). Since modules do not follow the taxonomic classification, they interacted with each other independent of their taxonomic status in this study. In addition, non-random associations were determined by the microbial physiological requirements and thus were more likely to be driven by the edaphic factors (Banerjee et al., 2016).

In this study, ST, SMC, as well as soil P and N concentrations showed the maximum number of associations and presented as the determinants for the seasonal dymatics of bacterial communities in the rhizospheric soil of *P*. *tabuliformis* on Qinling Mountains. Although some studies indicated the diversity and composition of soil bacteria community were significantly affected by pH variations (Kim et al., 2014), previous studies have shown that ST and SMC were the main influence factors to drive the activities and compositions of rhizospheric soil bacterial communities (Adams et al., 2010; Brockett et al., 2012). With regard to our study, most of the soil bacterial phyla were correlated with ST and SMC over the year, rather than the soil pH value. This might be due to the situ investigation in this study and minor changes in soil pH value among different seasons. Compared to other nutrients, furthermore, soil P and N concentrations showed more associations in the network analysis, suggesting the bacteria communities in rhizospheric soil of *P*. *tabuliformis* on Qinling Mountains were more susceptible to seasonal variations of soil P and N concentrations than other nutrients. Similar results were found from the study sites which characterized by temperate climate and plenty of plant litter and humus on the floor (DeForest et al., 2012; Zhang et al., 2013). Therefore, the soil bacterial communities would be significantly affected by soil P and N concentrations if there are large amount of organic matter and no organic carbon limiting in soil.

## Conclusion

In the present study, the effects of environmental factors on the rhizospheric soil bacterial communities of *P. tabulaeformis* forest on Qinling Mountains were analyzed and quantified during four different seasons. The results suggested that most of the edaphic properties varied along with seasonal changes and were influence the composition and structure of soil bacterial community. Biomass of the soil bacterial community prominently was increased in summer and autumn, but species richness and diversity were decreased. Microclimatic factors of ST and SMC, as well as the contents of soil nutrients N, P were the most important factors for driving the shift of soil bacterial community along with seasonal changes. The changes in the temporal pattern of the soil bacterial community regulated the enzymatic activities of soil urease and phosphatase, which in turn affected the cycling of soil nutrients N and P. Co-occurrence patterns of microorganisms in the rhizospheric soil provided a perspective on the bacterial community that the assembly of bacterial community was determined by the environmentally driven functional characteristics but not phylogeny. In a word, compared to other edaphic properties, ST, SMC, soil N and P exerted the strongest effect on the seasonal distribution of the bacterial community in *P*. *tabulaeformis* rhizospheric soil on Qinling Mountains, which was characterized by plenty of plant litter and without C-limiting. In the future, field investigations at greater latitude and altitude scales are needed to predict the climatic dynamic patterns of the bacterial assembly.

## Author Contributions

CW designed and supervised this study. MT and CS supervised this study. HW, HC, QD, and QX performed the experiments. HW refined the data analysis and writing.

## Conflict of Interest Statement

The authors declare that the research was conducted in the absence of any commercial or financial relationships that could be construed as a potential conflict of interest.

## Footnotes

1 http://drive5.com/uparse/

2 http://www.arb-silva.de

3 http://sourceforge.net/projects/rdp-classifier/
