# Supplementary material for: Phosphorus and Nitrogen Drive the Seasonal Dynamics of Bacterial Communities in Pinus Forest Rhizospheric Soil of the Qinling Mountains
Source: Front Microbiol. 2018 Aug 27;9:1930. doi: 10.3389/fmicb.2018.01930 (PMC6119707; doi:10.3389/fmicb.2018.01930)

Fig. S1: Linear regression relationships between TP/NH_4^+ and Alpha-diversity indices of CHAO, Simpson and Evenness.

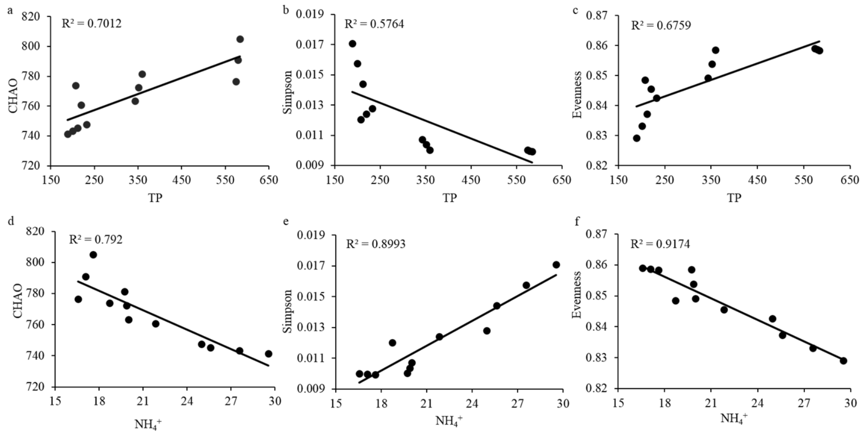

Supplement: Supplementary file 1 [file Image_1.PDF]
