# Supplementary material for: Phosphorus and Nitrogen Drive the Seasonal Dynamics of Bacterial Communities in Pinus Forest Rhizospheric Soil of the Qinling Mountains
Source: Front Microbiol. 2018 Aug 27;9:1930. doi: 10.3389/fmicb.2018.01930 (PMC6119707; doi:10.3389/fmicb.2018.01930)

Fig. S2: The differential genera between season from Kruskal-Wallis rank sum test. Red colored spots indicate genera with $P < 0.05$ and green colored spots indicate genera with $P \geq 0.05$.

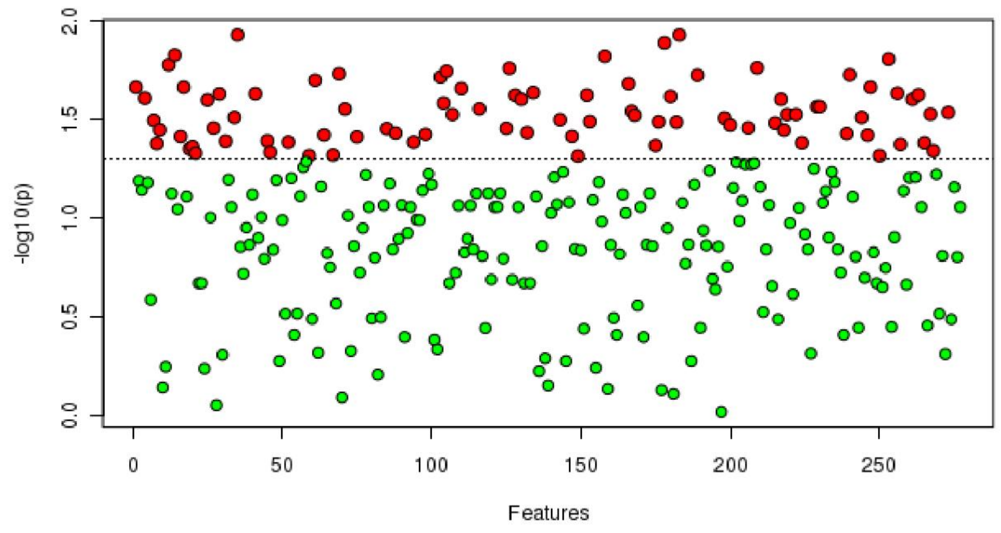

Supplement: Supplementary file 2 [file Image_2.PDF]
