# Supplementary material for: Phosphorus and Nitrogen Drive the Seasonal Dynamics of Bacterial Communities in Pinus Forest Rhizospheric Soil of the Qinling Mountains
Source: Front Microbiol. 2018 Aug 27;9:1930. doi: 10.3389/fmicb.2018.01930 (PMC6119707; doi:10.3389/fmicb.2018.01930)

Fig. S3: Results of MCODE analysis showing eight sub-networks with highest node scores. Networks and connectedness were visualized in CYTOSCAPE version 3.2.0 software. MCODE app was used to analyse networks with maximum scores.

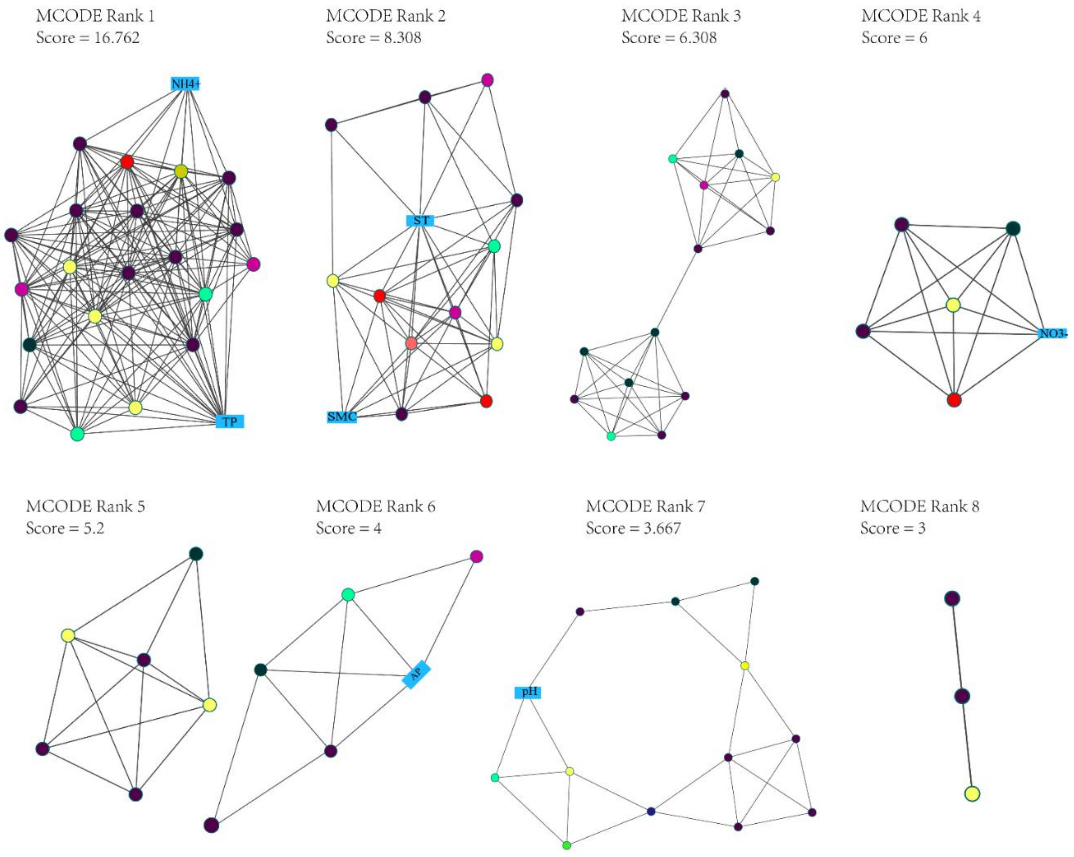

Supplement: Supplementary file 3 [file Image_3.PDF]
