# Supplementary material for: Phosphorus and Nitrogen Drive the Seasonal Dynamics of Bacterial Communities in Pinus Forest Rhizospheric Soil of the Qinling Mountains
Source: Front Microbiol. 2018 Aug 27;9:1930. doi: 10.3389/fmicb.2018.01930 (PMC6119707; doi:10.3389/fmicb.2018.01930)
Supplement: Supplementary file 4 [file Table_1.PDF]

Table S1. Spearman's correlation coefficients between Alpha-diversity metrics of bacterial community and biogeochemical characteristics of soil samples.

Variables	Shannon	Richness	Evenness
SMC	**-0.664 ***	-0.286	**-0.783 ****
pH	0.252	0.413	0.014
ST	**-0.628 ***	-0.370	**-0.716 ****
TK	0.266	0.314	0.056
TN	0.224	0.018	0.336
TP	**0.874 ****	0.533	**0.853 ****
AK	0.063	-0.180	0.238
AP	-0.503	-0.360	-0.371
NO_3^-	0.196	0.265	-0.035
NH_4^+	**-0.685 ***	**-0.614 ***	**-0.636 ***
SOM	0.196	-0.014	0.343
C/N	-0.035	0.039	-0.154

Data in bold indicate significant correlations, *: significant at $P < 0.05$; **: significant at $P < 0.01$; ***: significant at $P < 0.001$.
