# Supplementary material for: Phosphorus and Nitrogen Drive the Seasonal Dynamics of Bacterial Communities in Pinus Forest Rhizospheric Soil of the Qinling Mountains
Source: Front Microbiol. 2018 Aug 27;9:1930. doi: 10.3389/fmicb.2018.01930 (PMC6119707; doi:10.3389/fmicb.2018.01930)
Supplement: Supplementary file 5 [file Table_2.PDF]

Table S2. Mantel tests between soil properties, enzyme activities, alpha diversities and community composition across seasons.

	NH_4^+	ST	AP	TP	pH	SMC	Enzyme activity	Alpha diversity	Community composition
NH_4^+	1	-0.033	0.168	0.246	-0.036	0.278	0.320	0.231	0.179
	ST	1	-0.138	0.226	-0.046	0.087	0.171	-0.107	0.459**
		AP	1	0.529	0.496	0.356	0.399*	0.094	0.296*
			TP	1	0.699	0.091	0.428**	0.171	0.495**
				pH	1	-0.200	0.180	0.075	0.350*
					SMC	1	0.275	-0.099	0.239
						Enzyme activity	1	0.272	0.256*
							Alpha diversity	1	0.015
								Community composition	1

Data in bold indicate *r* value from Mantel test; *: P < 0.05; **: P < 0.01; ***: P < 0.001
